# Addressing Emotional Dysregulation Within NDBI for Young Autistic Children: Outcomes and Factors Related to Change

**DOI:** 10.3390/bs15070975

**Published:** 2025-07-17

**Authors:** Elizabeth H. Kushner, Chloe B. Holbrook, Nicole M. Hendrix, Josie Dylan Douglas-Brown, Katherine E. Pickard

**Affiliations:** 1 Department of Psychology, Emory University, 36 Eagle Row, Atlanta, GA 30322, USA; 2 Marcus Autism Center, Children’s Healthcare of Atlanta, 1920 Briarcliff Rd NE, Atlanta, GA 30329, USA; chloe.holbrook@choa.org (C.B.H.); nicole.m.hendrix@emory.edu (N.M.H.); josie.d.douglas-brown@emory.edu (J.D.D.-B.); katherine.e.pickard@emory.edu (K.E.P.); 3 Department of Pediatrics, School of Medicine, Emory University, 2015 Uppergate Dr, Atlanta, GA 30322, USA

**Keywords:** autism, early intervention, naturalistic developmental behavioral intervention, emotion regulation

## Abstract

Despite high rates of emotional dysregulation among autistic children, few studies have explored interventions addressing dysregulation. Naturalistic developmental behavioral interventions (NDBIs) are a class of interventions focused on supporting social communication. As social communication and emotion regulation skills emerge from similar developmental processes, NDBIs may be one approach for addressing dysregulation among autistic children. The present study sought to characterize change in dysregulation among one-hundred and eleven caregiver–child dyads completing Project ImPACT, a caregiver-mediated NDBI. Caregivers reported on child communication and social engagement using the Social Communication Checklist and emotion regulation using the Emotional Dysregulation Inventory-Young Child at the beginning and end of services. Clinicians reported on caregiver fidelity at each intervention session. Children showed reductions in emotional dysregulation throughout Project ImPACT, though reductions were specific to children who began the program with elevated dysregulation. Child social engagement at baseline and caregivers’ fidelity to specific strategies within Project ImPACT were associated with reductions in emotional dysregulation. Very few studies have tested interventions aimed at supporting emotion regulation among young autistic children. These findings demonstrate that NDBIs may support emotion regulation as well as social communication skills. Further incorporating support for emotion regulation in NDBI may address this critical gap without increasing service coordination for families.

## 1. Introduction

Emotion regulation (ER) is the ability to modify behavior and emotions in order to effectively solve problems to achieve goals (Menefee et al., 2022). An individual’s ability to recognize their emotional state, and thus, monitor their behavior, can have a cascading impact on their functioning (Menefee et al., 2022) and is related to school success, mental health outcomes, and overall well-being (Balzarotti et al., 2016; Graziano et al., 2007; Menefee et al., 2022). Emotional dysregulation has been widely studied across clinical populations, including among autistic individuals. Autistic individuals experience higher rates of internalizing and externalizing disorders and demonstrate fewer adaptive ER strategies compared to the general population (Berkovits et al., 2017; Mazefsky et al., 2014; Samson et al., 2014). Within a large study of children and adolescents (6–17 years of age), autistic individuals demonstrated significantly higher levels of emotional dysregulation compared to non-autistic individuals (Conner et al., 2021). A number of factors may predispose autistic individuals to elevated levels of emotional dysregulation, such as executive functioning and sensory processing differences (Faja & Dawson, 2015). Previous research has demonstrated that emotional dysregulation mediates the relationship between sensory dysregulation and challenging behaviors, suggesting that poorer emotion regulation may exacerbate some of the challenges associated with sensory processing differences, though these are separate constructs (Sung et al., 2024). Many studies have linked emotional dysregulation and higher rates of mental health conditions, including anxiety and depression, and psychiatric service utilization, making emotional dysregulation an important target for intervention (Conner et al., 2021; Reyes et al., 2019).

Studies suggest that differences in regulation among autistic children begin early in life (Jahromi et al., 2013; Samson et al., 2014). Toddlers with autism demonstrated a higher intensity of negative emotions within caregiver–child interactions and less frustration reduction when using age-appropriate self-regulation strategies (Day et al., 2022; Macari et al., 2017; Northrup et al., 2024). Research focused on validating a measure for emotional dysregulation in toddlers and preschoolers with and without autism showed that young autistic children demonstrated significantly higher levels of emotional reactivity (e.g., behavior such as outbursts or a quick onset of emotional responses) and emotional dysphoria (e.g., somewhat blunted emotional reactions), on average, compared to children without autism (Day et al., 2023).

While a number of studies have evaluated programs targeting ER in autistic adolescents and adults, few programs have been developed for toddlers and preschoolers (Conner et al., 2019; Shaffer et al., 2023; Factor et al., 2019). Indeed, a review of caregiver-mediated interventions for autistic children under 6 found that only two studies had measured ER within studies of caregiver-mediated interventions for young autistic children (Gulsrud et al., 2010; Hendrix et al., 2022; Rispoli et al., 2019). Gulsrud et al. (2010) found a reduction in negative affect and an increase in maternal emotional scaffolding following participation in a joint-engagement-focused caregiver-mediated intervention. Rispoli et al. (2019) piloted a caregiver-mediated intervention targeting emotional dysregulation among five young autistic children. The results demonstrated reductions in child dysregulation and increases in caregiver emotion coaching. 

Although few studies have explored interventions for ER among autistic children in early childhood, a range of evidence-based interventions have demonstrated efficacy in improving ER skills in a range of populations (Heron-Delaney et al., 2016; Rothenberg et al., 2019; Skowron & Funderburk, 2022). In the early years of development, parent-mediated interventions, which are designed to be implemented by caregivers with the support of professionals, are especially effective in reducing emotional dysregulation (Kennedy, 2020). Caregivers play a central role in the early development of emotion regulation. This process, often referred to as co-regulation, involves caregivers responding to and matching the emotional states and behaviors of their children (Cohn & Tronick, 1987). Initially, interaction is largely child-led, with caregivers responding to child behaviors. Caregivers’ responsiveness supports children in understanding the meaning of their own behavior and learning strategies to communicate, self-soothe, and regulate their own states (Aureli et al., 2022; Northrup & Iverson, 2020; Tamis-LeMonda et al., 2014). Broadly, the core components of parent-mediated interventions for ER skills are (1) strengthening the caregiver–child relationship by supporting caregiver sensitive responsiveness, reducing criticism and increasing warmth within interactions, and (2) improving parents’ ability to anticipate and respond effectively to children’s emotions and behaviors (Dozier et al., 2008; Eyberg & Robinson, 1982; Webster-Stratton, 1998). Research shows that the extent to which caregivers and infants are attuned to one another in interaction is related to infants’ developmental outcomes, including cognitive, language, and regulatory skills (Feldman, 2007). Given the role that these co-regulatory processes play across development domains, it follows that these strategies are incorporated within multiple programs targeting different populations. However, there is variation in the specific techniques taught to parents, and the way in which those techniques are taught (e.g., in vivo parent coaching and parent teaching/support).

Naturalistic developmental behavioral interventions (NDBIs) encompass a range of flexible interventions designed to meet the unique needs of autistic youth (Schreibman et al., 2015). While NDBIs specifically promote the development of social communication, play, and adaptive skills in autistic children, they share many features of existing ER interventions. Like many parent-mediated ER interventions, NDBIs are implemented (often by caregivers) within everyday contexts, fostering naturalistic learning opportunities and skill generalization. Caregivers are taught to center and follow children’s interests in both NDBIs and parent-mediated ER interventions, given the role of responsive caregiving practices in developing social communication and regulation skills (Feldman, 2007). Indeed, the developmental strategies included in NDBIs closely mirror the child-directed strategies included in well-researched parent-mediated ER interventions, such as Parent–Child Interaction Therapy (Eyberg & Robinson, 1982). Within NDBIs, caregivers follow their child’s lead to build social engagement and facilitate learning, whereas ER-focused interventions might use the same strategies to strengthen the bond between caregiver and child and support co-regulation. Although NDBIs do not explicitly include ER-related components, caregivers are coached to use their own affect to modulate their child’s engagement and regulation, and their flexibility allows for the incorporation of ER-related skills, such as emotion labeling, effective emotional expression, and the use of coping strategies (Kushner et al., 2024). A recent article showed that following participation in a parent-mediated NDBI program, children demonstrated reductions in anxiety and aggression (Liu et al., 2025). Thus, there are overlapping theoretical underpinnings and strategies between parent-mediated ER programs and NDBIs. Given the need to support multiple developmental areas, including social communication, language, and emotion regulation among young autistic children, NDBIs may be a particularly efficient approach for this population. However, given the historical focus on social communication in NDBI, additional research may be needed to understand whether and how NDBIs, as they are currently delivered, effectively target emotion regulation in addition to social communication.

Project ImPACT (Improving Parent as Communication Teachers; Ingersoll & Dvortcsak, 2010), an NDBI designed to support social engagement, communication, and play skills in autistic children, may be well positioned to have secondary impacts on emotional dysregulation. Project ImPACT comprises both developmental and behavioral strategies. Developmental strategies emphasize fostering strong parent–child relationships and promoting positive interactions. Parents receive coaching on their use of techniques like following their child’s lead and imitating their child to foster connection and communication. Behavioral strategies teach parents to create opportunities within motivating and engaging activities that encourage their child to use a new skill and to reinforce their child when the new skill is used (e.g., encouraging the use of a gesture to request rather than crying). Importantly, parents are explicitly coached to “shape their interaction” with their child so that it appropriately balances teaching and modeling new skills that are matched to their child’s motivation, regulation, and developmental level, supporting the development of foundational ER skills and reducing instances of emotion dysregulation. Further, as with other parent-mediated ER interventions, Project ImPACT provides parents with structured training, support, and coaching around behavior management techniques. Despite this overlap, few studies have focused on emotional dysregulation within the context of NDBI or tested the hypothesized relationships between NDBI strategies and ER skills.

Given the overlap in strategies between NDBIs and other caregiver-mediated interventions targeting ER, the present study aims to examine emotional dysregulation as an outcome of the NDBI Project ImPACT. This study has two aims. First, we will determine whether children participating in Project ImPACT demonstrate reductions in caregiver-reported emotional dysregulation from program entry to completion. Second, we aim to assess the factors associated with individual change in emotional dysregulation, including baseline social communication skills and caregivers’ use of specific Project ImPACT strategies. To address these questions, we will assess child factors, including child expressive communication, social engagement, and emotional dysregulation via caregiver report, before and after completing Project ImPACT. Additionally, clinicians will report on caregiver fidelity to each component of Project ImPACT to measure variation in the delivery of developmental, behavioral, and pacing strategies. We hypothesize that caregiver fidelity to developmental and pacing strategies will be most strongly related to change in child emotional reactivity. As discussed above, developmental strategies and strategies focused on shaping the interaction teach caregiver responsiveness and affective matching, which show strong relationships with child ER in the extant literature (Feldman, 2007).

## 2. Materials and Methods

### 2.1. Procedures

The present study is a secondary analysis of data collected routinely as a part of research conducted within an interdisciplinary outpatient clinic setting housed within a large children’s hospital. Participants seen within the clinic included young autistic children. All clinical services centered on the use of Project ImPACT and were delivered in a 12- to 14-week outpatient model (Ingersoll & Dvortcsak, 2019). Services were provided in-person within the clinic or via telemedicine using EPIC telehealth MyChart patient portal, a HIPAA-compliant video conferencing software. All families participating in early intervention services within this clinic are invited to opt into research alongside their routine services, or they can participate in services without completing research. Therefore, families included in the present study received the standard administration of Project ImPACT within a routine clinical setting. Families participating in research consent to the use of data collected for routine clinical purposes to be used in research. Measures of social communication and emotional dysregulation were administered at a family’s first and final session of the program. All data collection procedures were approved by the Children’s Healthcare of Atlanta Institutional Review Board (IRB).

### 2.2. Participants

Participants included 111 caregivers of autistic children between the ages of 14 and 48 months (M = 33.8; SD = 8.02) participating in routine early intervention services within a children’s hospital system. Child and caregiver demographic information is presented in Table 1. The present study included young autistic children and children with social communication delays who completed at least eight sessions of Project ImPACT within the clinic. This was chosen as the cutoff for completion, as caregivers completing the first eight sessions would have received all core components of the treatment, including developmental, behavioral, and pacing strategies.

### 2.3. Intervention

Project ImPACT is an evidence-based, caregiver-mediated NDBI with a focus on supporting social engagement, communication, imitation, and play skills using both developmental and behavioral strategies (Ingersoll & Dvortcsak, 2019). Project ImPACT is well researched and promotes caregiver use of program strategies, child social communication skills, and responsive caregiver–child interactions (Ingersoll et al., 2016; K. E. Pickard et al., 2016; Stahmer et al., 2020; Yoder et al., 2021). A family’s first Project ImPACT session focuses on goal setting to determine goals that are appropriate for a family’s priorities and a child’s current skill level. For example, if a child is using word approximations, an appropriate communication goal may be to support the use of full single words. The remainder of the sessions consist of a combination of didactics to teach caregivers Project ImPACT strategies and coach caregivers in the use of these skills as they apply to a child’s identified goals. Coaching occurs during naturalistic play and routines. The program begins with developmental strategies to build child engagement through positive and child-led interactions. Next, families begin learning how to incorporate behavioral strategies during moments of child engagement to support the development of new skills. Finally, families incorporate pacing strategies to ensure that they are able to balance these two sets of strategies during interaction. In the current study, Project ImPACT was delivered once per week for 1 h. Interventionists were licensed psychologists and speech–language pathologists certified in the delivery of Project ImPACT.

### 2.4. Measures

#### 2.4.1. Emotional Dysregulation Inventory-Young Child

Caregivers completed the Emotional Dysregulation Inventory-Young Child version (EDI-YC) at the beginning and end of their participation in Project ImPACT. The EDI-YC is a caregiver-reported measure validated for young children (2–5 years old) with and without autism (Day et al., 2023). Within this sample, a total of 9 children fell outside of the normative age range for the EDI-YC. Of these children, one was 14 months of age, and all of the others were between 20 and 24 months of age. The present study used the 15-item reactivity index, which is focused on capturing emotional reactivity. Items probe the frequency and intensity of children’s emotional reactions, for example, “Has explosive outburst” and “Difficult to distract if he/she is frustrated or upset.” Caregivers endorse the extent to which their children exhibit these behaviors, ranging from (1) Not at all: Never *happens* to (5) Severe: Almost always happens or causes a serious problem. The EDI-YC shows convergent validity with well-established caregiver report measures, such as the Child Behavior Checklist, and shows high internal consistency.

#### 2.4.2. Social Communication Checklist

Caregivers completed the Social Communication Checklist (SCC) upon Project ImPACT entry and completion. The SCC is a 70-item checklist that is broken down into five major domains: (1) social engagement; (2) expressive communication; (3) receptive communication; (4) imitation; and (5) play. Within each domain, items are listed in a developmental sequence and caregivers are asked to report whether their child engages in each skill: (1) rarely or not yet; (2) sometimes but not consistently; or (3) usually (at least 75% of the time). A total score is calculated by summing items across each domain, with a higher score indicating greater social communication skills. Potential scores for each domain range from 15 to 45. Previous research using the SCC has demonstrated its reliability, sensitivity to change within caregiver-mediated interventions, and association with other measures of social communication (Wainer et al., 2017). The present study focused on the social engagement and expressive communication domains of the SCC.

#### 2.4.3. Caregiver Fidelity

Following each session, clinicians rated the extent to which caregivers implemented intervention strategies with fidelity on a scale from 1 to 5. A score of 1 indicated that caregivers rarely used a strategy, and scores of 5 indicated that caregivers consistently implemented a strategy correctly. Clinicians rated caregivers’ fidelity in the five primary intervention strategies taught in Project ImPACT: follow your child’s lead, adjust your communication, create opportunities, teach new skills, and shape the interaction. A score of 4 or above indicates that a caregiver is implementing a strategy with fidelity. For behavioral definitions of each area of fidelity, see Ingersoll and Wainer (2013). In the present study, fidelity scores are derived from an average of fidelity ratings across all sessions a family completed within the outpatient clinic. Strategies were grouped as either “developmental” (i.e., strategies to focus on your child and adjust communication) or “behavioral” (i.e., strategies to create opportunities and teach new skills) by summing the average scores of each individual strategy. As “shape the interaction” can be implemented with both developmental and behavioral strategies, this skill was analyzed separately.

### 2.5. Data Analysis

First, we characterized child emotional reactivity at baseline by examining the average and spread of scores among a sample of children completing Project ImPACT. Next, we examined how emotional reactivity changed as children completed the program. We used a paired sample *t*-test to examine whether there was a change in emotional reactivity from baseline to completion of the intervention. Given the widespread scores within our sample and the potential for variation in change depending on children’s beginning levels of reactivity, we also split the full sample into two groups: those with baseline reactivity scores falling below the median and those falling above. Next, we conducted paired sample *t*-tests within each of these subgroups. Finally, we sought to determine whether baseline child characteristics and caregivers’ Project ImPACT strategy use predicted change in child emotional reactivity. To accomplish this, we used multiple linear regressions, where emotional reactivity was the outcome and child characteristics and baseline child emotional reactivity were predictor variables. To determine whether baseline child emotional reactivity interacted with child features, we also sought to assess whether there were interactions between group membership (high or low baseline reactivity) and predictor variables. Requisite assumptions for linear regression were not grossly violated in our data. All statistical analyses were conducted using R software (v4.4.3; R Foundation for Statistical Computing, 2024, Vienna, Austria).

## 3. Results

### 3.1. Reactivity at Baseline

Of the 111 included participants, the average total emotional reactivity score at baseline was 37.91 (*SD* = 15.47). The average score for our sample fell within the clinically significant range, or any raw score above 23 (Day et al., 2022). Therefore, the sample of children presenting for specialty parent-mediated NDBI clinical care demonstrated elevated dysregulation when compared to other same-aged autistic children. Baseline child emotional reactivity was not significantly correlated with age, baseline social engagement, or expressive communication measured using the SCC.

### 3.2. Change in Reactivity

**Figure 1:**
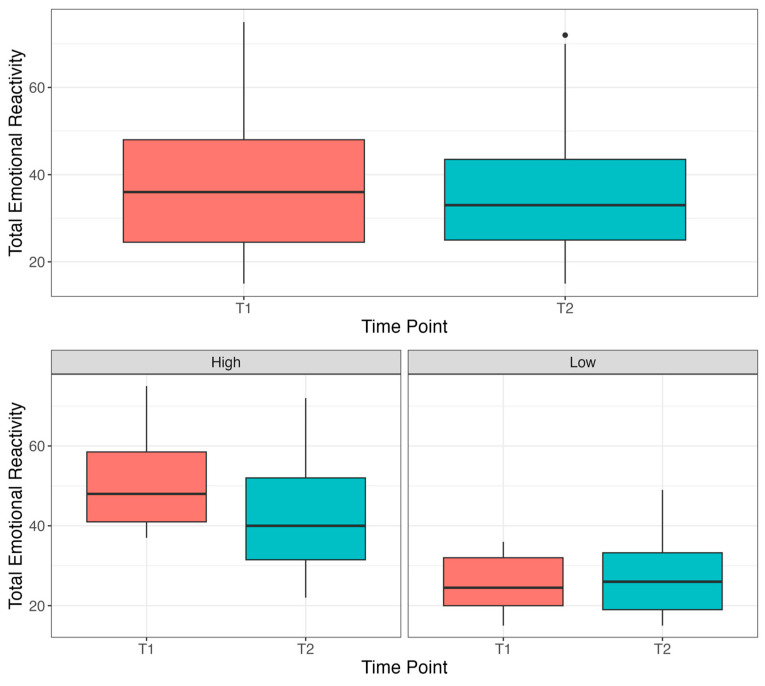
Emotional reactivity scores before and after participation in Project ImPACT.

The results of a paired sample *t*-test showed significant reductions in emotional reactivity measured on the EDI-YC after completing Project ImPACT (*t* (110) = 2.35, *p* = 0.02). Within the full sample, average reductions in the emotional reactivity were significant but small, on average 2.8 points on the EDI-YC, which accounts for less than one third of a standard deviation (see Figure 1). To assess whether baseline levels of emotional reactivity affected outcomes following intervention, the group was split at the median (*Mdn* = 36) into two groups reflecting “high” (baseline scores above the median) and “low” (baseline scores below the median) baseline reactivity. As shown in Figure 1, the “high” baseline reactivity group demonstrated significant reductions in reactivity from baseline to post-test (*t* (54) = 3.94, *p* < 0.001), demonstrating a 7.72-point reduction in emotional reactivity, which approaches one standard deviation. The “low” baseline reactivity group demonstrated trending but non-significant changes (*t* (55) = −1.73, *p* = 0.09).

Notably, baseline reactivity was the only variable of significant difference between these two groups at baseline. There were no differences between groups in child age or baseline expressive communication or social engagement (*p*’s > 0.05). Given the different patterns of intervention response between high- and low-reactivity groups, the following analyses included grouping as a term in all regression models.

### 3.3. Relationship Between Child Characteristics and Emotional Reactivity Outcomes

**Figure 2:**
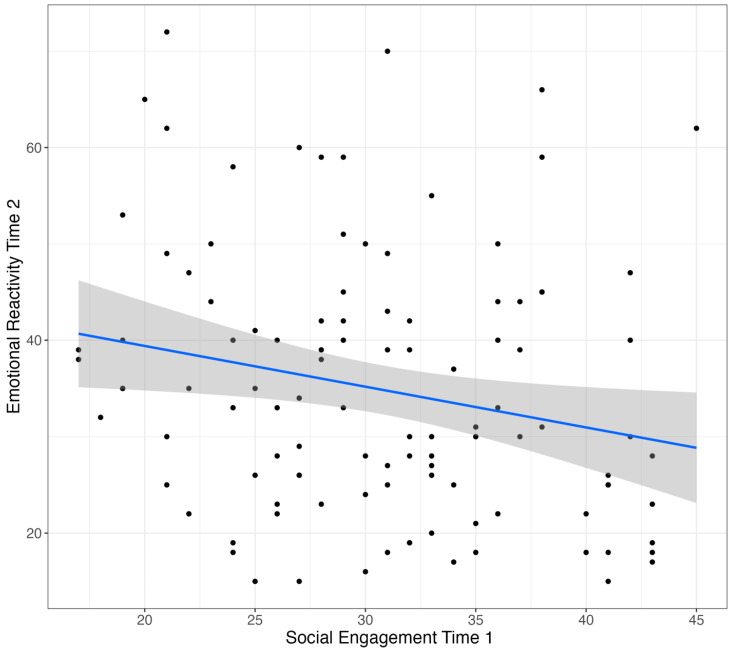
Social engagement at baseline and emotional reactivity at post-test. **Note**. Dots represent individual data points and gray shaded area represented 95% confidence interval for the regression line.

In order to examine the effects of baseline child characteristics on change in emotional reactivity, we assessed the relationship between child social engagement and expressive communication skills at the beginning of the intervention and emotional reactivity at the end. To examine these relationships, multiple regression was used to test the main effects of child characteristics and group membership (high or low baseline emotional reactivity), as well as an interaction between the two on emotional reactivity at post-intervention. Reactivity at baseline was included to control for initial levels of reactivity. The results show that baseline child social engagement significantly predicted emotional reactivity at intervention completion when controlling for baseline emotional reactivity, indicating that higher social engagement at the beginning of intervention predicted lower reactivity scores at outcome (*β* = −0.37, *p* < 0.01; Figure 2). There was no significant main effect of or interaction with group membership, indicating that this association was the same for children with low and high baseline reactivity levels. There were no significant effects in a model examining the relationship between baseline child expressive communication and emotional reactivity following intervention, meaning there was no relationship between a child’s expressive communication skills and change in reactivity throughout the intervention.

### 3.4. Associations Between Caregiver Project ImPACT Fidelity and ER Outcomes

In order to examine which elements of Project ImPACT might be associated with changes in emotional reactivity, we assessed the relationship between emotional reactivity and caregiver fidelity to developmental, behavioral, and pacing intervention strategies. We utilized four separate multiple regression models to assess the associations between each of these fidelity domains and emotional reactivity and its effects on the outcome. Notably, fidelity was not scored for all participants due to changes in clinic procedures; therefore, these analyses were conducted with a sub-sample (*n* = 63). As in previous analyses, additional predictors included baseline emotional reactivity scores to control for variation in baseline reactivity and the interaction between baseline reactivity (membership in the “high”- or “low”-reactivity groups split at the median) to test whether associations between fidelity and change in reactivity varied based on initial levels of reactivity. For the results of all regression models, see Table 2. First, overall caregiver fidelity to program strategies was significantly associated with reductions in child emotional reactivity while controlling for baseline reactivity. There was a significant main effect of total fidelity to program strategies on emotional reactivity at post-test (*β* = −2.22, *p* = 0.02), while the effects of the groups and the interaction between the groups and fidelity were not significant. Caregiver fidelity to developmental strategies was not related to emotional reactivity at intervention completion, nor did this relationship vary across group membership. In other words, within these analyses, caregivers’ fidelity to developmental strategies was not associated with change in child emotional reactivity.

**Figure 3:**
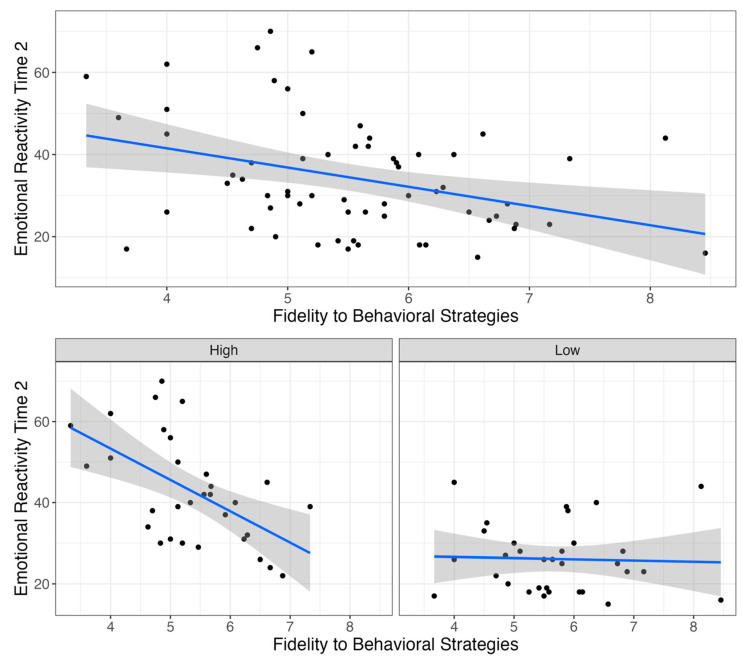
Caregivers’ use of behavioral strategies and emotional reactivity outcome. **Note**. Dots represent individual data points and gray shaded area represented 95% confidence interval for the regression line.

**Figure 4:**
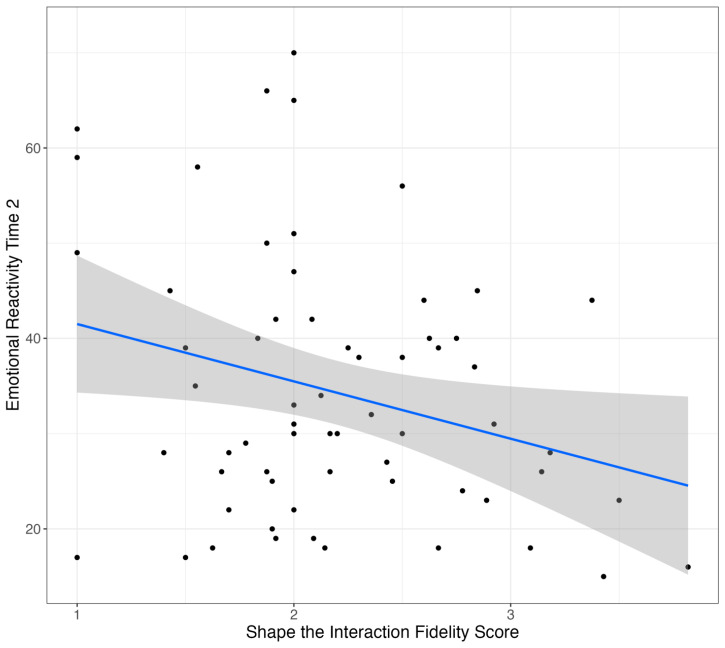
Caregivers’ use of the “shape the interaction” strategy and emotional reactivity outcomes. **Note**. Dots represent individual data points and gray shaded area represented 95% confidence interval for the regression line.

In a model examining associations between caregiver fidelity to behavioral strategies and emotional reactivity following Project ImPACT, there was a significant main effect of fidelity to behavioral strategies (*β* = −6.31, *p* = 0.001), a significant main effect of “low” group membership (*β* = −35.98, *p* = 0.02), and a significant interaction between fidelity to behavioral strategies and group membership (*β* = 5.5, *p* = 0.03) on emotional reactivity at post-intervention. Thus, depending on group membership or the level of emotional reactivity at baseline, there were different relationships between caregiver fidelity to behavioral strategies and change in child reactivity. As shown in Figure 3, there was a relationship between behavioral strategy use and emotional reactivity, specifically for children with high baseline reactivity. For children with high baseline reactivity, higher caregiver fidelity to behavioral strategies predicted lower emotional reactivity, whereas there was no relationship between these variables among children with low baseline reactivity. Finally, there was a significant main effect of fidelity to “shape the interaction” or the pacing of the interaction, on emotional reactivity at post-intervention, such that higher fidelity was related to greater reductions in reactivity for the full sample (*β* = −8.41, *p* = 0.02; Figure 4).

Finally, we assessed the relationship between caregivers’ implementation of developmental and behavioral strategies in Project ImPACT. The results of a paired samples *t*-test showed that there was significantly higher average fidelity to developmental strategies (*M* = 7.29, *SD* = 1.06) than behavioral (*M* = 5.53, *SD* = 1.03) strategies in our sample (*t* (62) = 15.77, *p* < 0.001). Further, fidelity to developmental strategies was significantly correlated with fidelity to behavioral strategies within our sample (*r* = 0.64, *p* < 0.001). These correlations are similar when looking only within high (*r* = 0.64, *p* < 0.001) and low (*r* = 0.67, *p* < 0.001) reactivity groupings.

Taken together, these findings suggest that improvements in core Project ImPACT domains are related to reductions in child emotional reactivity across intervention, though different skills may be supporting ER depending on the nature and severity of dysregulation at baseline. Additionally, clinician-rated caregiver fidelity to program strategies was significantly associated with reductions in child emotional reactivity.

## 4. Discussion

The present study examined ER as an outcome of the NDBI Project ImPACT, as well as factors associated with change in emotional reactivity. First, the results demonstrated that there was a high prevalence of clinically significant emotional dysregulation within the outpatient sample. Additionally, there were significant reductions in emotional reactivity from baseline to the completion of Project ImPACT when delivered in routine outpatient services. Further examination revealed that this change was driven by children who began the program with elevated levels of emotional reactivity. Analyses testing associations between emotional reactivity and core social communication targets of NDBI showed that baseline social engagement significantly predicted reductions in emotional reactivity. Ratings of caregiver fidelity, or the extent to which caregivers implemented Project ImPACT strategies during their sessions, were also significantly associated with reductions in emotional reactivity. When breaking caregiver fidelity into developmental, behavioral, and pacing strategies, only fidelity to behavioral and pacing strategies significantly predicted reductions in emotional reactivity, though developmental and behavioral strategies were highly correlated. Taken together, these findings indicate that NDBIs may be a promising avenue for addressing emotional dysregulation in young autistic children.

The results showed a high prevalence of clinically significant emotional reactivity among a group of autistic children participating in outpatient parent-mediated NDBI services. This prevalence has implications for practice; for example, there may be a greater need to support early intervention providers in addressing dysregulation. Within state-funded early intervention systems, providers describe adapting sessions to address family needs not included in primary content, for example, to address emotional dysregulation (K. Pickard et al., 2023). Given that most NDBIs do not currently explicitly integrate this content, these adaptations are left to the discretion of the provider. A diverse group of professionals are involved in implementing early interventions for young autistic children, including speech–language pathologists, psychologists, and special education professionals, among others (Ingersoll et al., 2024). Professionals report varying degrees of experience and comfort directly discussing behavior and regulation with families (Kushner et al., 2024). Given the prevalence of emotional dysregulation among autistic children presenting for a parent-mediated intervention, it may be beneficial to more directly integrate content on ER into NDBI frameworks and incorporate these strategies into provider training.

### 4.1. Change in Emotional Dysregulation Throughout Project ImPACT

Our results demonstrate that children showed reductions in emotional reactivity throughout the course of Project ImPACT. This finding is consistent with recent work showing reductions in anxiety and aggression among young autistic children following participation in the Early Start Denver Model, another NDBI (Liu et al., 2025). The ability to address ER within NDBI may be a promising approach for several reasons. As discussed, NDBIs share many features with existing programs designed to address emotional dysregulation among young children (Dozier et al., 2008; Eyberg & Robinson, 1982). Therefore, it may be possible to build on existing content to support regulation within the NDBI framework. Given the increasing use of NDBI programs within state-funded early intervention services, it may be advantageous to address dysregulation within the context of NDBI rather than within an additional program. Addressing multiple targets within a single program may also alleviate some stress for caregivers, as caregivers of autistic children often engage in and coordinate many services which has been linked to caregiver well-being (Hodgetts et al., 2015, 2017). Therefore, addressing multiple goals within NDBIs, when appropriate, may both effectively support regulation for autistic children and alleviate service inefficiency and service coordination needs.

### 4.2. Factors Associated with Change: Caregiver Fidelity and Child Characteristics

The results show that the components of Project ImPACT most associated with reductions in child emotional reactivity were behavioral and pacing strategies. It could be that behavioral strategies that coach caregivers to support children in using new communication to request, protest, and advocate result in reduced dysregulation, and that caregiver responsiveness to new communication is meaningful and regulating. We originally hypothesized that developmental and pacing strategies would be most strongly associated with improvements in emotional reactivity due to a large body of work demonstrating the relationship between responsive caregiving and child regulation (Feldman et al., 1999; Wass et al., 2024). Though we did not observe a relationship between fidelity to developmental strategies and improved child regulation, our results suggest that caregivers with the highest behavioral fidelity may have had the highest overall fidelity to Project ImPACT strategies. One hypothesis for this result was that, generally, caregivers may have had higher and less variable fidelity to developmental strategies than behavioral strategies, as behavioral strategies can be more complex to implement and are introduced later in the program. It may be that caregivers who had higher fidelity to behavioral strategies also had higher fidelity to developmental strategies, indicating that to best target reactivity, strategies must be used in tandem.

Pacing strategies, or the balance of developmental and behavioral skills within interaction, were the only elements of Project ImPACT that predicted improvements in regulation for the full group. Previous qualitative studies report that clinicians describe using pacing strategies within Project ImPACT to explain and coach around co-regulation in sessions (Kushner et al., 2024). Therefore, these scores likely reflect the extent to which a caregiver accurately reads and responds to their child’s signals and readiness to learn in a given moment. This is consistent with the previous literature on co-regulation, which generally refers to a caregiver’s ability to match and respond to their child’s state and indicates that these behaviors support child emotion regulation (Silkenbeumer et al., 2016). These findings suggest that existing NDBI content may improve child emotion regulation and lend support for addressing regulation within this framework. Further developing coaching around pacing as it applies to emotion regulation may be a promising area on which to build in training new clinicians to support regulation within Project ImPACT and other NDBIs.

The present study identified two child characteristics associated with greater improvements in emotional reactivity throughout Project ImPACT. First, children who began the program with levels of emotional reactivity above the group median showed reductions throughout the program. Given that these sessions were conducted in routine clinical services, clinicians were able to adapt manualized content to address the needs of a specific child and family. Therefore, it is possible that clinicians more directly supported caregivers with regulation-focused strategies or applied Project ImPACT strategies to regulation-related goals when a child’s level of dysregulation was high, for example, by providing coaching around how to regulate a caregiver’s own affect and activity level to adjust that of their child during moments of dysregulation. Indeed, clinicians report primarily addressing dysregulation within NDBI sessions if it is interfering with the delivery of the program or families advocate for its inclusion as a goal (Kushner et al., 2024). It is also possible that caregivers of children with higher baseline dysregulation were more motivated to execute strategies and apply them to regulation-related goals during interaction. Second, baseline levels of child social engagement, as reported by caregivers, were associated with improvements in regulation throughout Project ImPACT. Studies show that co-regulation is facilitated by coordinated engagement between caregivers and children within interaction (Feldman et al., 2011). Autistic children engage in less joint engagement during interaction with caregivers, which may have cascading effects on the development of ER (Adamson et al., 2009; Guo et al., 2017). Within Project ImPACT, children who begin with higher levels of social engagement may have access to more opportunities for co-regulation during joint play with caregivers, and therefore, make greater improvements in regulation. Future research might explore whether children with high levels of dysregulation benefit from greater time spent on content related to social engagement or whether a certain threshold of engagement is needed before children can benefit from other NDBI strategies.

### 4.3. Strengths and Limitations

A strength of this paper is the collection of data within routine clinical services in a clinic serving a diverse population. Conducting research within this setting allows us to speak to the potential of NDBIs for addressing dysregulation not only within lab-based trials but also within sustainable, routine clinical services. While this is a strength of the study, this approach also leads to some limitations. For example, participating families are not randomized. This design limits the ability to ascribe causality in the relationships between caregiver fidelity and changes in child dysregulation. Additionally, as families are aware of their participation in the intervention, there is a possibility that this can affect their reporting on child social communication and ER following the intervention. Relatedly, the present study used only caregiver report measures for assessing child dysregulation and social communication. While these measures have strong properties and are associated with more objective measures of these constructs, it is always possible that other caregiver perceptions and biases contribute to child ratings, which can influence the results. Additionally, though all families completed at least 75% of all Project ImPACT sessions and would have been exposed to all core program material, there may have been some variation in the number of sessions each family completed. This also reflects the routine clinical setting in which this research was conducted, and generally, future studies should replicate and extend these findings in a more standardized treatment setting. Finally, though clinicians implementing Project ImPACT train to reliability with one another, fidelity ratings included in the present study were not co-coded, and therefore, we cannot present interrater reliability information on the presented fidelity ratings.

## 5. Conclusions

The present study explores emotional reactivity as an outcome of Project ImPACT, a widely studied and implemented NDBI program. Supporting ER within these programs may address dysregulation without the demand of additional time and resources for families and children. Future studies will be needed to determine for which children NDBIs might be an appropriate approach to addressing dysregulation and what additional elements may be important to incorporate. Further, given the diverse workforce implementing NDBIs and variation in training backgrounds, future work should consider the best training approaches to ensure all clinicians have the tools to address regulation when appropriate. Taken together, these results suggest that NDBIs may be a promising approach to supporting this critical skill for autistic children.

## Figures and Tables

**Table 1:** Early intervention participant demographics (*n* = 111).

	Mean	SD	% (*n*)
Age (months)	33.80	8.02	
Average household income by zip code	USD 78,400	USD 16,600	
Sex			
Male			69.37 (77)
Female			22.52 (25)
Unknown/declined to report			8.11 (9)
Ethnicity			
Hispanic/Latino			7.21 (8)
Non-Hispanic			72.97 (81)
Unknown/declined to report			19.82 (22)
Race			
African American/Black			39.64 (44)
Asian			0.90 (1)
Biracial/multiracial			2.70 (3)
White/Caucasian			36.94 (41)
Unknown/declined to report			19.82 (22)

**Table 2:** Multiple regression results for caregiver Project ImPACT fidelity scores and change in emotional reactivity.

Model	*b*	95% CI [LL, UL]
**Total Fidelity**		
Intercept	54.24 **	[18.3, 90.14]
Baseline Emotional Reactivity	0.43 *	[0.10, 0.75]
Total Fidelity	−2.22 *	[−4.06, −0.37]
Group: Low	−32.49	[−70.6, 5.62]
Fidelity Total × Group: Low	1.82	[−0.48, 4.11]
**Fidelity: Developmental Strategies**		
Intercept	25.67	[−11.29, 63.13]
Baseline Emotional Reactivity	0.52 **	[0.19, 0.85]
Developmental Strategies	−1.19	[−5.22, 2.83]
Group: Low	−3.45	[−44.1, 37.2]
Developmental Strategies × Group: Low	0.01	[−5.12, 5.14]
**Fidelity: Behavioral Strategies**		
Intercept	56.97 ***	[27.69, 86.24]
Baseline Emotional Reactivity	0.39 *	[0.08, 0.7]
Behavioral Strategies	−6.31 **	[−10.11, −2.51]
Group: Low	−35.98 *	[−67.18, −4.79]
Behavioral Strategies × Group: Low	5.53 *	[0.54, 10.53]
**Fidelity: Shape the Interaction**		
Intercept	37.25 **	[13.24, 61.27]
Baseline Emotional Reactivity	0.46 **	[0.15, 0.77]
Shape the Interaction	−8.41 *	[−15.45, −1.38]
Group: Low	−19.6	[−42.13, 2.93]
Shape the Interaction × Group: Low	7.2	[−1.47, 15.88]

Note. Significant at the * <0.05, ** <0.01, *** <0.001 level.

## Data Availability

Data are available upon request.

## Abbreviations

The following abbreviations are used in this manuscript:

NDBI	Naturalistic Developmental Behavioral Intervention
ER	Emotion regulation
EDI-YC	Emotion Dysregulation Inventory-Young Child
SCC	Social Communication Checklist
